# Secondary immune-mediated thrombotic thrombocytopenic purpura in idiopathic inflammatory myopathy: a case-based review

**DOI:** 10.1007/s00296-022-05260-8

**Published:** 2022-12-28

**Authors:** Nikolas Ruffer, Marie-Therese Holzer, Lukas Can Bal, Simon Melderis, Martin Krusche, Tobias B. Huber, Ina Kötter

**Affiliations:** 1 III. Department of Medicine, University Medical Center Hamburg-Eppendorf, Martinistraße 52, 20246 Hamburg, Germany; 2 Department of Rheumatology and Immunology, Klinikum Bad Bramstedt, Bad Bramstedt, Germany

**Keywords:** Antisynthetase syndrome, Myositis, Thrombotic microangiopathy, Thrombocytopenia, *ADAMTS13 *protein

## Abstract

Immune-mediated thrombotic thrombocytopenic purpura (iTTP) is a potentially fatal acquired thrombotic microangiopathy syndrome that frequently develops in the context of infectious diseases or systemic autoimmune conditions including connective tissue diseases. We report the case of a 42-year-old female suffering from severe iTTP associated with anti-Jo-1 positive antisynthetase syndrome, which was successfully treated with combination therapy of intravenous immune globulin, rituximab and plasma exchange. Based on a systematic review of the literature, two additional cases of idiopathic inflammatory myopathy-associated iTTP (secondary iTTP) were identified. In conclusion, iTTP may be a rare complication of IIM that clinicians should consider in cases of marked thrombocytopenia. Further work-up of this finding should include a peripheral blood smear (schistocyte count) and *ADAMTS13* activity. The concomitant manifestation of these autoimmune conditions may require intensive immunosuppressive therapy.

## Introduction

Immune-mediated thrombotic thrombocytopenic purpura (iTTP) is a potentially fatal acquired thrombotic microangiopathy (TMA) syndrome that is strongly associated with autoantibodies targeting *ADAMTS13* (A Disintegrin and Metalloprotease with ThromboSpondin type 1 motif, member 13) leading to impaired cleavage of von Willebrand factor (vWF) multimers with subsequent formation of platelet-rich microthrombi [1, 2]. TMA syndromes frequently (~ 50%) develop in the context of infectious diseases or systemic autoimmune conditions including connective tissue disease (CTD) such as systemic lupus erythematosus (SLE) [3]. Interestingly, iTTP has also been reported as a rare complication in idiopathic inflammatory myopathies (IIM) and circulating myositis-specific antibodies (MSA) have been implicated in the pathogenesis of both disorders [4, 5].

Here, we report a rare case of severe secondary iTTP associated with anti-Jo-1 positive antisynthetase syndrome (ASyS), which was successfully treated with intravenous immune globulin (IVIG) therapy, rituximab (RTX) and plasma exchange (PEX).

Since the clinical features and treatment of iTTP in IIM have not been systematically investigated, a systematic review of the literature was conducted. Thus, the aims of this work were (1) to report our own experience with this subject and (2) to identify and analyze previously published cases of iTTP in IIM.

## Case report

**Fig. 1:**
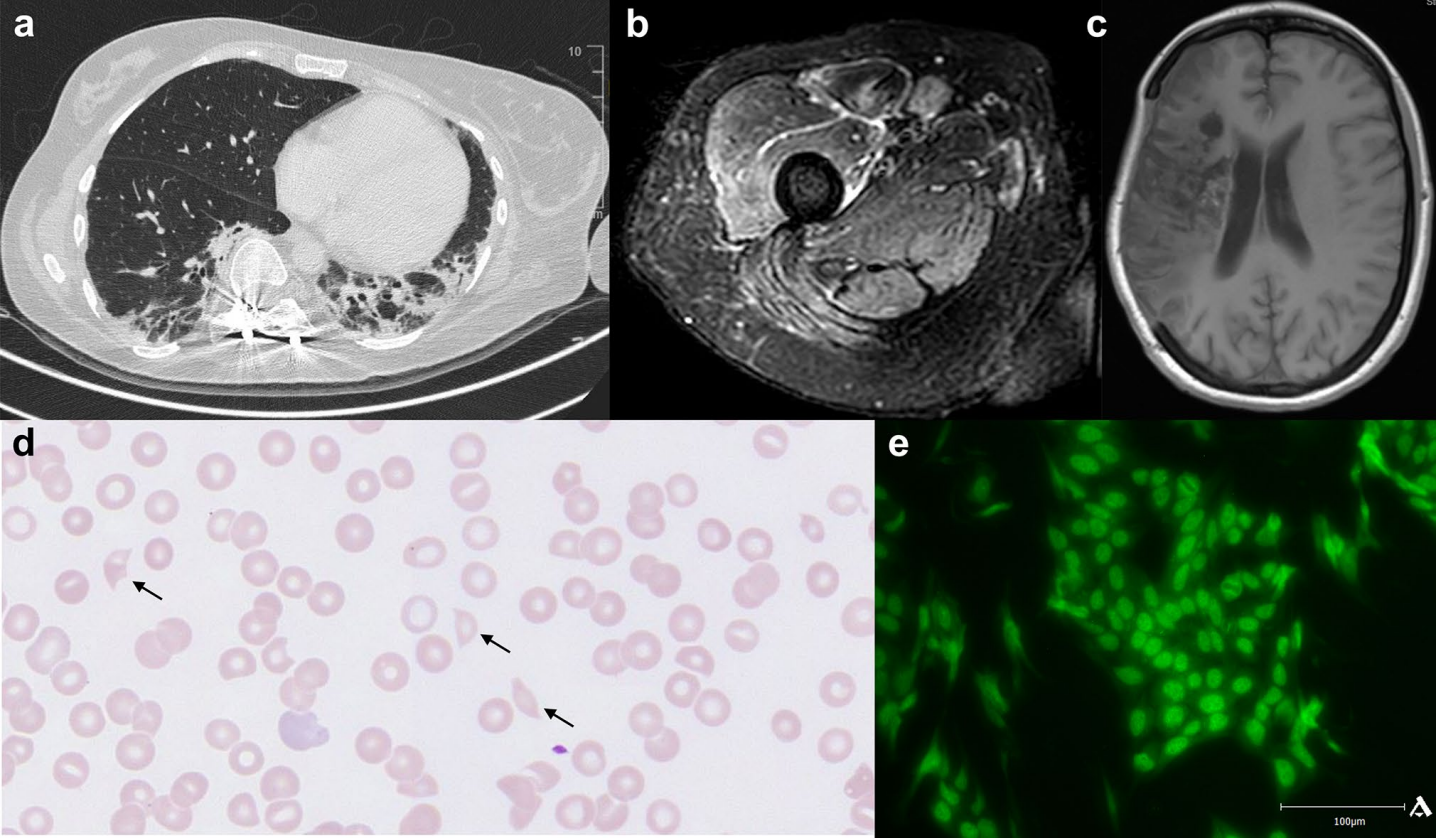
**a** Computed tomography of the chest (transversal) demonstrating bilateral pulmonary infiltrates consistent with alveolitis. Note: The patient also suffered from concomitant scoliosis treated with spinal fusion. **b** Magnetic resonance imaging (transversal, T2-weighted Dixon Turbo Spin Echo sequence) of the right thigh consistent with myositis. **c** Magnetic resonance imaging (transversal, T1-weighted FL2D sequence) of the brain indicating ischemia of the right middle cerebral artery. **d** Peripheral blood smear demonstrating schistocytes (black arrows). **e** Immunofluorescence staining (patient sample) of anti-Jo-1 antibodies in antisynthetase syndrome

A 42-year-old female with marked thrombocytopenia, anemia and elevated inflammatory markers without response to antibiotic, prednisolone pulse (500 mg/day for 3 days) and IVIG (40 g) therapy was referred to our department. Two months earlier, she had suffered an ischemic stroke (Fig. 1c) due to tandem occlusion of the right internal carotid artery and right cerebral medial artery (M1 segment). At that time, angiography of the cerebral vessels did not show any signs of vasculitis or small vessel disease and the complete blood count was normal. Thorough history and clinical examination at that time did not suggest the presence of CTD, while antinuclear antibodies were found. However, the patient reported a history of scoliosis treated with spinal fusion.

Physical examination on admission revealed unchanged left hemiplegia but no signs of fever or skin changes. The complete blood count showed bicytopenia with thrombocytopenia (platelet count 30 000/µL, ref. 150 000–450 000/µL) and normochromic normocytic anemia (hemoglobin 9.1 g/dL, ref. 12–16 g/dL). Schistocytes (1%, ref. < 0.1%) were seen on a peripheral blood smear (Fig. 1d). Further laboratory work-up was notable for elevated levels of C-reactive protein (114 mg/L, ref. < 5 mg/L), creatine kinase (331 U/L, ref < 145 U/L), lactate dehydrogenase (LDH; 810 U/L, ref. 125–248 U/L) and troponin (150 pg/mL, ref. < 15,6 pg/mL). Of note, urine analysis, creatinine and haptoglobin levels were normal. An autoimmune evaluation showed antinuclear (1:1280, AC-4 pattern), anti-Jo-1 (Fig. 1e) and anti-Ro52 antibodies. Microbiological investigations for virus infections (SARS-CoV-2, HBV, HCV, HIV, CMV, EBV), tuberculosis (interferon gamma release assay) and bacteremia (blood cultures) were negative. A bone marrow biopsy showed no evidence of hematologic malignancy. A full-body PET scan exhibited bilateral basal pulmonary infiltrates (Fig. 1a). Subsequent bronchoalveolar lavage exposed granulocytic alveolitis but no evidence of an infectious cause of the infiltrates. Magnetic resonance imaging of the non-plegic right thigh revealed edematous muscle changes indicating myositis (Fig. 1b). Therefore, anti-Jo-1 positive AS was diagnosed and treatment with IVIG 40 g/day for 3 days was administered, which subsequently led to an increase of the platelet count (148 000/µL).

**Fig. 2:**
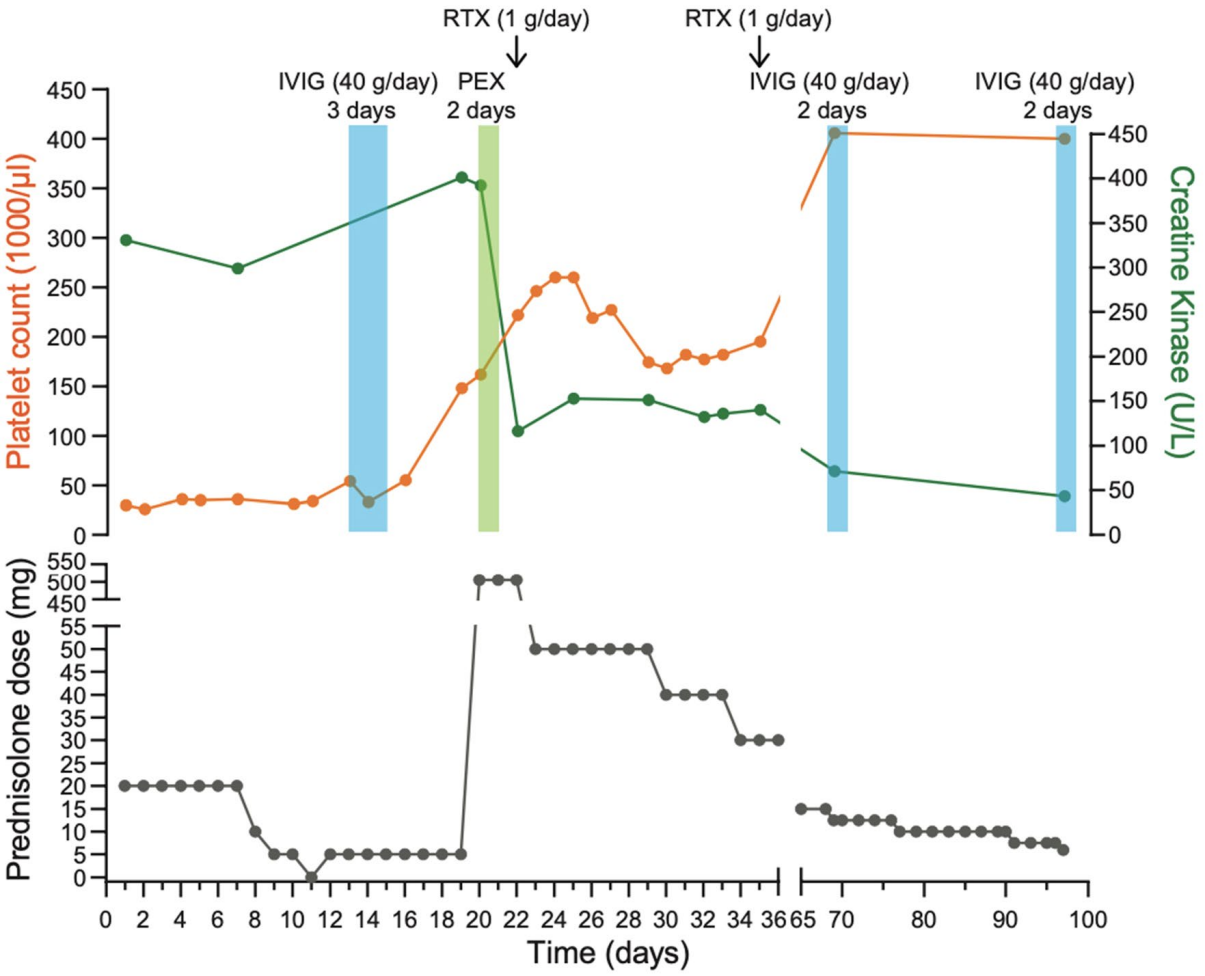
Laboratory parameters and treatment over the disease course: platelet count (orange), creatine kinase (green), and prednisolone dose (gray). Abbreviations: rituximab (RTX), intravenous immune globulin (IVIG), and plasma exchange (PEX)

Further work-up revealed suppressed *ADAMTS13* activity (< 0.01 U/ml, ref. 0.4–1.3 U/ml) due to the presence of inhibitory *ADAMTS13* antibodies (70 U/l, ref. < 12 U/l). In conclusion, secondary iTTP was diagnosed and PEX (2 consecutive days) accompanied by iv prednisolone (500 mg/day) for 3 days was started. Afterward, the patient received the first dose of RTX 1000 mg. Under this treatment, the platelet count normalized and caplacizumab was, therefore, not administered. Moreover, CK, CRP, LDH, troponin and schistocyte count all improved (Fig. 2). After 3 months of follow-up, platelet count and activity parameters of iTTP (hemoglobin, LDH, schistocytes) and ASyS remained normal during ongoing IVIG therapy and prednisolone 5 mg per day.

## Methods

Two databases (*MEDLINE* via PubMed, *Embase* via Ovid) were searched on 17/07/2022 to investigate the association between TTP and IIM. Keywords to identify publications related to IIM were ‘myositis’, ‘dermatomyositis’, ‘polymyositis’, ‘myopathy’, ‘antisynthetase syndrome’ and ‘Jo-1’. Keywords to identify publication related to TTP were ‘thrombotic thrombocytopenic purpura’, ‘thrombotic microangiopathy’, ‘Moschcowitz’ and ‘ADAMTS13’. Both components were used to search title and abstract of references (database search). The references of included publications were additionally screened for suitable records (references screening).

The retrieved records were imported to Rayyan [6] and independently screened by three of the authors (NR, LCB and M-TH) based on title and abstract. If titles and/or abstracts did not provide sufficient data for a decision, full texts were reviewed instead.

Case reports or case series reporting iTTP in the context of IIM were included for further analysis (Table 1). The following inclusion criteria were applied: (a) study published in a peer-reviewed journal; (b) publication in English language; (c) study type: case report or case series (individual data of reported cases available); (d) established diagnosis of IIM; (e) iTTP as defined by microangiopathic hemolytic anemia, thrombocytopenia and autoantibody-mediated *ADAMTS13* deficiency. The following exclusion criteria were applied: (a) records: conference abstracts or presentations; (b) language: other than English.

**Table 1:** Inclusion and exclusion criteria to identify cases of idiopathic inflammatory myopathy with concomitant immune-mediated thrombotic thrombocytopenic purpura from the literature

Inclusion criteria	Exclusion criteria
(a) Records: study published in peer-reviewed journal (b) Language: English (c) Study type: case report or case series with individual description of reported cases (d) Clinical case: idiopathic inflammatory myopathy with concomitant immune-mediated thrombotic thrombocytopenic purpura	(a) Records: conference abstract or presentation (b) Language: other than English

## Results

**Fig. 3:**
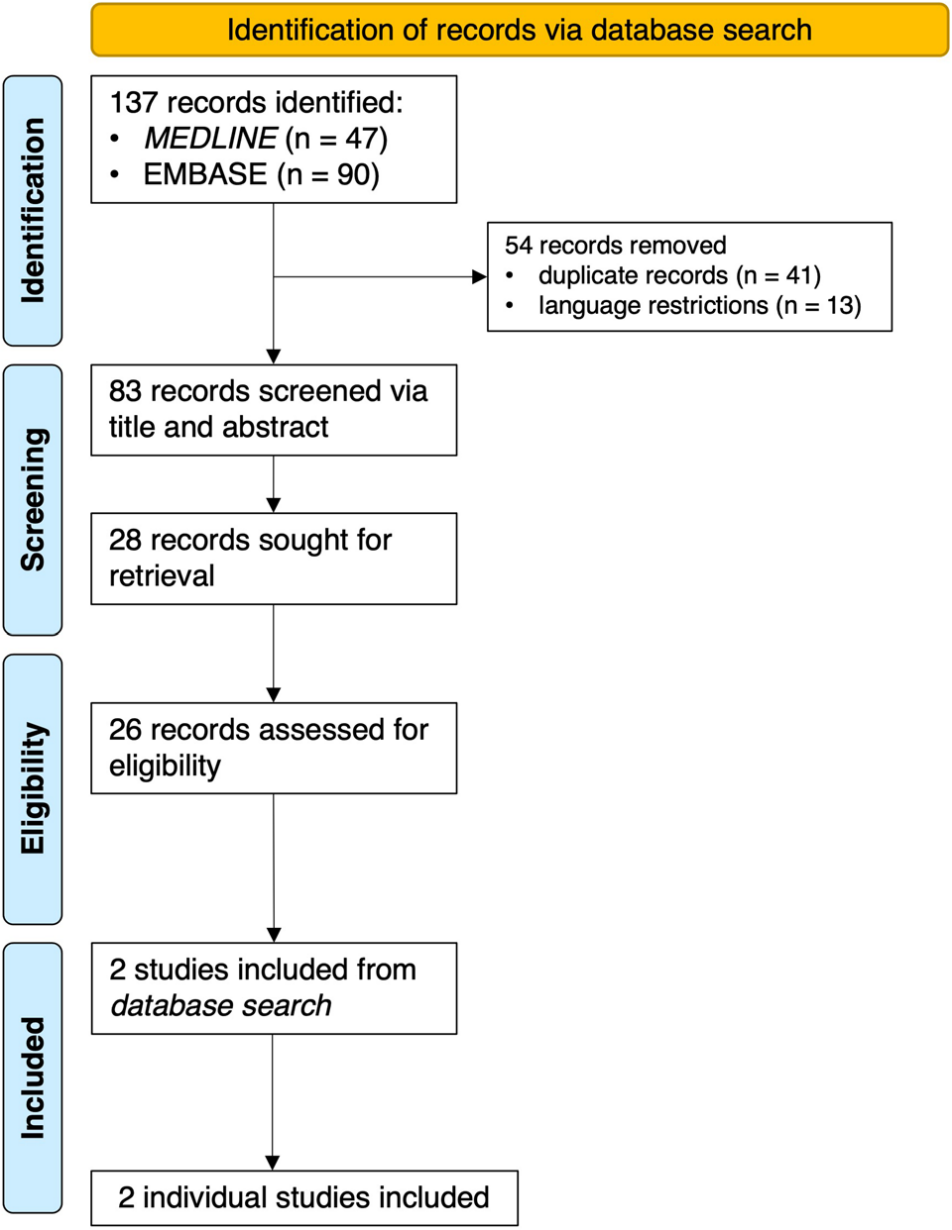
Flowchart detailing the selection process of included publications

The search strategy described above identified 137 publications of which 41 duplicates were removed. In total, 2 publications reporting 2 additional cases of IIM-associated iTTP were included in this study (Fig. 3, Table 2).

**Table 2:** Cases of idiopathic inflammatory myopathy-associated immune-mediated thrombotic thrombocytopenic purpura

Case	IIM	Clinical features	Laboratory	Treatment	Outcome
present case 42 y female	ASyS Jo-1 + Ro52 +	Interstitial lung disease Myositis Arthritis Cerebral ischemia	Hemolytic anemia Thrombocytopenia Schistocytes CRP 114 mg/l CK 313 U/l *ADAMTS13* deficiency *ADAMTS13* auto-ab +	IVIG PEX RTX GC	Remission of iTTP and ASyS
Goreshnik et al. [5] 59 y male	ASyS Jo-1 + Ro52 +	Interstitial lung disease Myositis Arthritis Fever	Hemolytic anemia Thrombocytopenia Schistocytes CK 7892 U/l *ADAMTS13* activity 11% *ADAMTS13* inhibitor +	PEX GC RTX MMF IVIG AZA	Good response
Yamada et al. [4] 75 y male	Paraneoplastic DM TIF1-γ +	Gastric cancer Tuberculosis Myelodysplastic syndrome	Hemolytic anemia Thrombocytopenia Schistocytes *ADAMTS13* deficiency *ADAMTS13* auto-ab +	PEX GC IVIG RTX	Death (septic shock)

*ASyS* antisynthetase syndrome, *AZA* azathioprine, *GC* glucocorticoids, *DM* dermatomyositis, *IVIG* intravenous immune globulin therapy, *RTX* rituximab, *PEX* plasma exchange, *MMF* mycophenolate mofetil, *y* years, +  = positive

Goreshnik et al. [5] described a 59-year-old male suffering from refractory ASyS with pulmonary involvement and relapsing secondary iTTP, who received high-dose prednisolone combined with B-cell (RTX)/antibody-directed therapies (PEX). Persistent CK elevation suggesting ongoing disease activity was finally treated with the addition of azathioprine and IVIG and resulted in a good response.

Yamada et al. [4] reported a 75-year-old male with paraneoplastic dermatomyositis, who developed an acute flare of secondary iTTP in the context of a *Mycobacterium tuberculosis* infection and ultimately died due to septic shock.

## Discussion

Acquired *ADAMTS13* deficiency due to immunological loss of self-tolerance is the hallmark of iTTP. Anti-*ADAMTS13* immunoglobulin G (IgG) autoantibodies can be detected in ~ 75% of cases and autoantibody-mediated inhibition of *ADAMTS13* activity represents the most common mechanism of action (via neutralizing action or acceleration of clearance) [2]. Specifically, almost all autoantibodies (~ 95%) recognize the spacer domain of *ADAMTS13* [2]. Other disease mechanisms such as the formation of immune complexes have also been described [2]. Importantly, *ADAMTS13* deficiency is the only known specific pathogenic factor for TTP, but it is not sufficient to induce active disease on its own and yet unidentified factors must be present [2]. TTP frequently develops in the setting of inflammatory conditions [2, 3].

Interestingly, an association between *ADAMTS13* biology and systemic autoimmune conditions has been reported in the literature: On the one hand, immune-mediated *ADAMTS13* deficiency has been described as a rare complication of various autoimmune conditions such as SLE [7, 8]. In one study, severe deficiency was more frequent in rheumatoid arthritis and SLE compared to systemic sclerosis and myositis [7]. On the other hand, non-inhibitory anti-*ADAMTS13* antibodies and low protease activity can be detected in SLE and systemic sclerosis patients without active iTTP [9]. CTD-TMA syndromes with normal *ADAMTS13* activity were also reported implicating other pathogenic factors [7]. Finally, anti-*ADAMTS13* antibodies without *ADAMTS13* deficiency can also be present in healthy donors [10].

The presence of MSA is strongly associated with distinct IIM phenotypes [11]. ASyS represents a subtype that is characterized by autoantibodies targeting aminoacyl transfer ribonucleic acid synthetases, myositis, interstitial lung disease and arthritis. Multiple studies indicate a possible role of anti-Jo-1 autoantibodies in the pathogenesis of ASyS [12]. For example, a correlation between anti-Jo-1 antibody levels and disease severity has been reported [13, 14] suggesting B-cell/antibody-directed treatment approaches for ASyS to reduce circulating autoantibodies.

So far, only one detailed report of a 59-year-old male with secondary iTTP and concomitant anti-Jo-1 ASyS has been published [5]. However, diagnostic criteria of TTP and IIM have been revised during the last decades and similar cases might be underreported [5]. Nevertheless, multiple cases of TMA associated with dermatomyositis and polymyositis have been described [4].

The patient described in our report suffered from severe iTTP associated with anti-Jo-1 positive ASyS affecting muscles and lungs. Interestingly, laboratory investigations at the time when cerebral ischemia developed showed a normal platelet count, inflammatory markers and CK values. Whether cerebral ischemia may be attributed to iTTP remains elusive.

Of note, repeated laboratory investigations showed normal serum haptoglobin concentrations in our patient despite active iTTP with anemia and thrombocytopenia. This finding is exceptionally unusual and urges clinicians to consider atypical presentations of TTP in the context autoimmune diseases.

As reported in the previous case of ASyS-associated iTTP, our patient received intensive immunosuppression for iTTP including PEX, glucocorticoids and RTX. The letter ones have also shown sufficient efficacy in AS [15–17]. This dual management addressed both antibody-mediated diseases present in our case. PEX has not been systematically investigated in ASyS, but clinical improvement has been reported in combination with immunosuppressive drugs in some cases of severe ILD [18]. IVIG therapy, in our case introduced for myositis, has also been reported to be effective iTTP [19] as demonstrated by our case. Specifically, an inhibitory effect on *ADAMTS13* antibodies by immunoglobulins is discussed.

In conclusion, our review supports the view that iTTP may be a rare complication in IIM, which clinicians should consider in cases of marked thrombocytopenia despite normal haptoglobin concentrations. Further work-up of this finding should include a peripheral blood smear (schistocyte count) and *ADAMTS13* activity. The combination of IVIG, PEX and RTX therapy may be considered to achieve remission in severe cases with life- and organ-threatening manifestations.

## Data Availability

The data underlying this article cannot be shared publicly due to the anonymization of the patient and for the privacy of individuals that participated in this work. Non-confidential data will be shared on reasonable request to the corresponding author.
